# The role of Iran's context for the development of health technology assessment: challenges and solutions

**DOI:** 10.1186/s13561-023-00438-7

**Published:** 2023-04-20

**Authors:** Meysam Behzadifar, Masoud Behzadifar, Maryam Saran, Saeed Shahabi, Ahad Bakhtiari, Samad Azari, Nicola Luigi Bragazzi

**Affiliations:** 1 Social Determinants of Health Research Center, Lorestan University of Medical Sciences, Khorramabad, Iran; 2 Health Policy Research Center, Institute of Health, Shiraz University of Medical Sciences, Shiraz, Iran; 3 Health Equity Research Center (HERC), Tehran University of Medical Sciences (TUMS), Tehran, Iran; 4 Research Center for Emergency and Disaster Resilience, Red Crescent Society of the Islamic Republic of Iran, Tehran, Iran; 5 Human Nutrition Unit Department of Food and Drugs, University of Parma Medical School, Building C, Via Volturno, 39, 43125 Parma, Italy

**Keywords:** Health technology assessment, Iran, Health policy, Healthcare systems

## Abstract

Health technology assessment (HTA) is a comprehensive and structured evaluation that aims to analyze the potential impacts of health technologies, including medical devices, diagnostic tools, pharmaceuticals, and public health interventions. Its purpose is to provide policymakers with evidence-based information to inform decisions related to the utilization and implementation of these technologies. HTA allows for the comparison of various scenarios related to a technology across a wide range of factors. This can aid in the creation of an essential drug list and health benefits package that is tailored to the actual needs of the community within a given healthcare system. In the present paper, we review the role of Iran's context for the development of HTA, in terms of challenges and solutions.

## Background

Health technology assessment (HTA) is a comprehensive and structured evaluation that aims to analyze the potential impacts of health technologies, including medical devices, diagnostic tools, pharmaceuticals, and public health interventions [1]. Its purpose is to provide policymakers with evidence-based information to inform decisions related to the utilization and implementation of these technologies [2]. HTA allows for the comparison of various scenarios related to a technology across a wide range of factors. This can aid in the creation of an essential drug list and health benefits package that is tailored to the actual needs of the community within a given healthcare system [3].

With increasing demand for efficient and safe healthcare, many countries have turned to HTA as a means of improving the allocation of limited resources and maximizing their healthcare systems [4]. HTA has thus garnered the attention of policymakers at various levels of the healthcare system. As a result, institutionalizing HTA has become necessary for achieving universal health coverage (UHC) goals [5]. UHC aims to provide effective healthcare services to all members of the community, regardless of financial constraints, and the development and implementation of HTA can play a critical role in achieving this objective [6]. Many countries have already begun implementing HTA as a significant effort towards achieving their UHC goals [7]. Policymakers can leverage HTA to inform their decisions regarding health system priorities, and to identify interventions that provide the greatest benefit to the health system [8]. In situations where budgetary constraints are a reality, setting priorities in healthcare is crucial to meet societal needs [9]. HTA can serve as a powerful tool for countries to ensure sustainable resource allocation toward achieving UHC [10]. Inefficiencies in resource allocation and weaknesses in core health system functions result in the waste of significant financial resources in healthcare systems across all economic statuses [3]. This issue is particularly pressing for LMICs, which have committed to increasing investment in essential health services and responding to the needs of their communities [11]. As a result, many LMICs have prioritized the development and implementation of HTA to determine their healthcare priorities [3]. The relationship between health policy and HTA is essential, as the latter provides valuable information for the allocation of limited resources to address real needs and promote access and fairness in health service delivery [12].

## Main text

### The emergence of HTA

The HTA field was established in the 1970s at the request of the Congress in the United States. Following this development, wealthy and developed countries became interested in HTA as a means of managing the costs of their health systems and responding to the needs of their covered populations. In Europe, the development and implementation of HTA began in the 1990s. Sweden was the first country to establish an agency related to HTA in 1987, followed by Canada. Australia also began its activities in the field of HTA in the early 1990s. The UK and Germany started their HTA activities in 1999 and 2004, respectively [3]. The implementation and development of HTA is rapidly taking shape in many countries. Countries such as China, Thailand, Vietnam, Colombia, and Brazil are successful examples of developing nations where the health system can utilize HTA to obtain evidence for decision-making.

### Iran’s health system

Iran's healthcare system is a blend of public and private healthcare services. The majority of healthcare services in Iran are provided by the government [13]. According to the Ministry of Health and Medical Education (MoHME), 80% of the country's population cannot afford the tariffs charged by the private sector for healthcare services [14]. Supplementary insurance enables only 17% of the population to access private healthcare services and a mere 3% of Iranians utilize healthcare services provided by the private sector [15].

Primary healthcare (PHC) services in Iran are provided through a network of healthcare centers that offer basic healthcare services to the public [16]. These centers are located in both urban and rural areas and are operated by the MoHME. The primary healthcare services provided in these centers include preventive and curative care, maternal and child health services, family planning, immunization, health education, and disease control [17]. The healthcare centers are staffed by general practitioners, nurses, midwives, and public health experts. In addition to these centers, rural health houses provide basic healthcare services to rural populations [18]. The Iranian government has made significant efforts to expand access to primary healthcare services and improve the quality of care provided in these centers, particularly in rural and underserved areas [19]. The goal is to ensure that all citizens have access to basic healthcare services and to reduce the burden on secondary and tertiary healthcare facilities [20]. Over the past few decades, Iran's PHC system has made significant progress with improved access to healthcare, increased life expectancy, and a reduction in infant mortality rates [17].

The country's healthcare infrastructure comprises over 800 public hospitals, 200 private hospitals, and more than 22,000 primary healthcare centers [13]. To improve the quality of healthcare services and ensure universal access to healthcare for all citizens, the government has implemented various healthcare reforms, including expanding health insurance coverage to larger portions of the population, increasing the number of healthcare facilities, and investing in medical education and research [21]. The health transformation plan (HTP) was one of the most significant reforms implemented in Iran's healthcare system [22]. The goal of the policy was to improve the quality of healthcare services provided to the Iranian population and to increase access to healthcare, particularly for those in rural and underserved areas. Under the HTP, a number of measures were taken to improve the healthcare system, including [23, 24]:

- Expanding health insurance coverage: The government expanded the health insurance coverage to cover larger parts of the population, particularly those who were previously uninsured or underinsured. This enabled more people to access healthcare services.
- Increasing the number of healthcare facilities: The government invested in building new hospitals and healthcare centers, particularly in underserved areas, to increase access to healthcare services.
- Improving the quality of healthcare services: The government implemented measures to improve the quality of healthcare services, such as increasing the number of healthcare professionals and investing in medical education and research.
- Reducing healthcare costs: The government implemented measures to reduce the cost of healthcare services, such as controlling the prices of medical supplies and equipment.

The HTP has had a positive impact on the Iranian healthcare system, resulting in improved access to healthcare services and better healthcare outcomes for the population [25]. The HTP in Iran is closely tied to the goal of achieving UHC for the population [26]. The HTP has made significant strides towards achieving UHC by expanding health insurance coverage, increasing the number of healthcare facilities, and improving the quality of healthcare services [27]. Expanding health insurance coverage has been a crucial component of the health transformation plan [28]. The government has increased the number of people covered by the public health insurance system and introduced supplementary insurance plans for those who can afford to pay more for healthcare services [29]. These measures have helped reduce the financial burden on patients and ensure that more people have access to healthcare services [30]. In 2019, Iran allocated 6.71% of its GDP to healthcare [31]. During the same year, around 39.49% of healthcare expenses were paid out-of-pocket, which is higher than the global average of 18.1% [32]. The Iranian healthcare system has made significant progress in expanding insurance coverage to its citizens. As of 2021, over 70% of the Iranian population is covered by health insurance. The government has announced plans to increase this coverage to about 90% of the population [33].

The healthcare system in Iran is predominantly centralized, with the MoHME playing a crucial role in the stewardship, governance, management and administration of healthcare services [34]. The MoHME regulates the healthcare sector, sets healthcare policies and standards, oversees the allocation of resources, and manages healthcare facilities, including hospitals and clinics [14]. The MoHME operates healthcare facilities at the national level and collaborates with provincial and local health authorities to provide healthcare services throughout the country [13].

### Introduction and current scenario of HTA in Iran

In October 2007, an unit consisting of a team of experts began operating for the first time in the health promotion and network development center at the MoHME of Iran [35]. Initially, the unit evaluated a number of technologies such as Positron Emission Tomography (PET) scan and Hyperbaric Oxygen Therapy (HBOT). However, following structural changes within the MoHME, the HTA unit's activity was transferred to the deputy of treatment, and the health technology evaluation office was established, which had a more comprehensive and structured approach [36]. Since 2010, the office has been formally carrying out HTA activities at the national level, with an increase in the number of technology evaluations and efforts to develop it at the local level. In the same year, in collaboration with the National Institute of Health Research (NIHR), the HTA-related research was assigned to the institute's researchers through a call [37]. The researchers then submitted the final reports to the HTA office [38]. This process continues, and every year, the HTA office prepares reports on the number of evaluations conducted, which are handed over to the NIHR. After being approved by HTA experts in Iran, these reports are then given to policymakers for decision-making [35].

Additionally, since 2009, one university began accepting and training students in the HTA master's course, and this process has continued. Currently, four universities accept students annually in the HTA field, and researchers have been trained in this area [39]. Furthermore, some medical science universities have recruited researchers in this field. The development of evidence-based decision-making, use of local data for HTA, holding courses and workshops to enhance researchers' skills, helping to develop and launch HTA in medical science universities, and planning to conduct HTA research and attracting the participation of other organizations for the development of HTA are some of the office's goals in Iran [40].

### Challenges for development and implementing HTA in Iran

There are numerous challenges involved in the development and implementation of HTA in Iran. These challenges have direct and indirect impacts on HTA considering Iran's circumstances. However, four major challenges stand out: political capacity building, cultural frameworks, economic issues, and insufficient data.

The first challenge, the issue of political capacity building, is primarily related to political factors and the influence of policymakers. Robust HTA frameworks require significant financial and human resources. Policymakers may hesitate to invest in the development of HTA, especially when other policies are prioritized for funding. In Iran, HTA is not a priority for many policymakers as they tend to favor policies that yield short-term results and are not willing to take the risk of investing in policies such as HTA, which will have long-term consequences. Some Iranian policymakers, based on their political ideology, are opposed to limiting people's access to new technologies and are unwilling to tolerate protests against non-use of these technologies, even if they may not be cost-effective for the healthcare system. However, many policymakers may prioritize cost containment over patient access to medical technology. The conflict between these two views can make it difficult to build a consensus on the goals and objectives of HTA. In recent years, the equipment industry and pharmaceutical companies in Iran have shown resistance to certain budget control policies, and this can be a controversial issue. The causes of these resistances may start with lobbying from powerful political sources, which can hinder the development of HTA. Perhaps one of the most significant challenges is the lack of sufficient awareness of the nature of HTA among policymakers. Public perception of HTA is essential, and the lack of awareness and understanding of HTA can be influenced by media coverage, interest groups, and political messages. Negative perceptions of HTA can make it difficult to develop and implement evidence-based policies. In Iran, like many countries in the Middle East, people have a lot of respect for political officials, and there is a possibility that the society will show resistance to HTA due to the negative perceptions of policymakers towards HTA and evidence-based research.

The second issue is cultural frameworks because different cultures may have varying attitudes towards health, healthcare, and medical technologies. Cultural diversity and the presence of different ethnicities in Iran can lead to different perceptions of health and disease, as well as different definitions of what constitutes health and disease, and different attitudes towards medical interventions. Cultural differences can influence the development of HTA frameworks as assessments may need to consider cultural variations in disease prevalence, treatment preferences, and healthcare utilization. In Iran, different cultures may have different value systems, which can affect the way healthcare resources are allocated. These differences can influence the development of HTA frameworks, as they may require balancing competing values and preferences. Clear communication and understanding of technical terms and concepts are essential for HTA frameworks. For example, traditional treatment methods may be preferred over evidence-based interventions. This could influence the development of HTA frameworks, as they may need to account for cultural variations in treatment preferences and healthcare utilization. Different cultures may have varying expectations and priorities regarding the allocation of healthcare resources. For instance, some cultures may prioritize providing basic healthcare services over costly medical interventions. In many provinces of Iran, cultural differences arise from the economic situation of the province, causing many people to prioritize their current expenses over spending on their health. For this reason, they may tend to use newer technologies, even though they are no more effective than other interventions.

The third issue that should be considered is Iran's economic challenges, which are related to the costs associated with the development and implementation of HTA, as well as the potential impact of HTA on healthcare systems and the broader economy. Developing and implementing HTA can be expensive and require significant resources and expertise, creating economic challenges for healthcare systems, especially in low-resource settings or where funding for HTA is limited. Implementing HTA recommendations can also have significant cost implications for healthcare systems, particularly if the recommended technologies are expensive or require significant infrastructure or training. Economic evaluation is an important part of HTA, involving the cost-effectiveness of healthcare interventions. However, conducting economic evaluations can be complex and resource-intensive, especially where limited data is available or the interventions being evaluated are complex. HTA frameworks should consider equity to ensure the distribution of healthcare resources is fair and equitable, but balancing equity considerations with economic considerations can be challenging, especially where limited resources are available for healthcare. HTA frameworks can also influence innovation, creating barriers to the adoption of new healthcare technologies due to time-consuming and costly evaluations. In addition, sanctions on Iran over the past two decades have significantly reduced the financial resources of the health sector, with many international organizations refusing to give financial aid. This has impacted the development and implementation of HTA in Iran, with reduced international participation in joint research and a lack of access to experiences in some technologies, resulting in significant research gaps.

The fourth issue is insufficient data in Iran's health system. The lack of transparent data and sufficient electronic databases and infrastructure for the development of HTA in Iran is a major challenge. Researchers need national and local data to conduct HTA studies and evaluate new interventions. In recent years, different governments have intended to launch the format of electronic file and digitalize various services of healthcare, but this has not been realized yet. The poor status of medical records and the lack of use of digital and mobile platforms to monitor and manage health services processes in Iran have resulted in inadequate data about many diseases and patients. Consequently, researchers face difficulties in conducting HTA evaluations and preparing credible evidence. The health record in Iran is incomplete, and some electronic structures of services in PHC and Health Information System (HIS) services in hospitals have not been fully implemented.

### Suggestions for future growth of HTA in Iran

To accelerate the innovation of HTA in Iran, several solutions can be implemented. Educating policymakers about the benefits of HTA and how it can help them make informed decisions regarding the allocation of healthcare resources can be helpful in raising their awareness. This can be achieved through holding workshops, short courses, brochures, and other educational materials. The fact that the MoHME operates as the trustee of HTA in Iran can be viewed as both an opportunity and a threat [41]. Currently, all HTA programs and policies in Iran are developed from top to bottom, without the involvement of many stakeholders. To develop HTA further, it is recommended to involve stakeholders such as patients, healthcare providers, and industry representatives in the HTA process, which can help build consensus on the need for HTA and gain the support of policymakers [3]. It is important to highlight the economic benefits of HTA, emphasizing cost savings and improved efficiency in the use of healthcare resources. Providing examples of successful HTA programs in other countries and highlighting the positive impact it has had on healthcare outcomes can help build confidence in the effectiveness of HTA as a tool to improve healthcare [4]. Additionally, any concerns or objections that policymakers may have about HTA, such as concerns about cost or the impact on innovation, should be addressed. Providing evidence-based answers to these concerns can help reduce fears and build support for HTA [42].

Interacting with people in the community to understand their values, beliefs, and attitudes towards health technologies and healthcare is crucial. Community participation can help identify cultural barriers and facilitators to HTA implementation, ensuring that HTA strategies are tailored to the needs and expectations of different communities [38]. Cultural perspectives should be considered in HTA frameworks, including the inclusion of traditional and complementary medicine, which may be widely used in some cultures [43]. Cultural beliefs and preferences of different ethnic groups in relation to health technologies and healthcare should also be taken into account [44]. The use of culturally appropriate language and terminology in HTA reports and communication materials can improve understanding and acceptance of HTA among different communities. Additionally, HTA frameworks should be accessible to people of different literacy levels and language abilities. Collaborating with cultural elites such as community leaders, religious figures, and healthcare providers who possess cultural knowledge and expertise, can facilitate communication and understanding between HTA experts and different communities [3]. This can also ensure that HTA strategies are culturally appropriate and acceptable, which in turn can influence policymakers to support HTA development. Moreover, HTA frameworks should prioritize equity and consider the impact of HTA on different cultural groups, including ensuring that HTA strategies do not exacerbate existing health inequities and address the needs of disadvantaged and marginalized communities. Addressing cultural mistrust and ensuring that HTA processes are transparent and inclusive can help build trust and acceptance of HTA among community members [5].

In addressing economic challenges, it is essential to prioritize HTA assessments based on their potential impact on health outcomes and cost-effectiveness [45]. This can help allocate resources more efficiently, ensuring that the most important assessments are done first [46]. Collaboration between stakeholders, including policymakers, healthcare providers, patients, and the industry, can help share costs and resources for HTA development and implementation [47]. Additionally, maximizing the use of existing data sources, such as administrative databases and electronic health records can reduce the need for costly primary data collection and analysis [48]. Standardization of HTA methods and processes in different regions or countries can reduce the cost and complexity of conducting assessments while also improving the comparability of results [46]. Value-based pricing models can be utilized to ensure that health technologies are priced based on their actual value to patients and the healthcare system, promoting cost-effective use of resources and reducing costs. Moreover, partnerships between public and private entities can facilitate the development and implementation of HTA as well as the translation of research findings into clinical practice [3].

To address the challenge of insufficient data for HTA development, it is important to invest in data infrastructure and standardize data collection processes across different healthcare providers [49]. As the quality and completeness of collected data improves, this will facilitate the development of HTA as the main infrastructure for policy makers' decisions in all aspects of the health system [50]. Encouraging data sharing between different healthcare providers and institutions can create a more comprehensive dataset for HTA development, which can be facilitated through legal and technical frameworks as well as incentives for data sharing [36]. Additionally, advanced analytical tools such as artificial intelligence can help identify patterns and trends in health care utilization and outcomes, allowing for more accurate and comprehensive HTA studies [51]. Using existing data sources, such as electronic health records, administrative databases, and national health surveys, can provide valuable information for HTA studies and can be more cost-effective than collecting new data [6]. Prioritizing the use of data for HTA development and decision-making can help ensure effective and efficient use of resources. This can be achieved by creating a culture of data use in the health care system and ensuring that HTA studies inform health policy and resource allocation decisions [10].

## Conclusion

Health Technology Assessment (HTA) is still in its early stages of development in Iran, and policymakers must leverage it to facilitate informed decision-making in healthcare. However, increasing political capacity, taking into account cultural frameworks and economic factors, and addressing insufficient data are significant challenges in advancing HTA in Iran. To enhance political capacity, policymakers need education on the advantages of HTA and its role in allocating healthcare resources. Moreover, various entities such as patients, healthcare providers, and industry representatives should be involved in HTA, taking into account cultural frameworks and economic factors. Sufficient availability of data, such as electronic health records, administrative databases, and national health surveys, can be utilized to evaluate HTA. Collaboration among all stakeholders is crucial for the development of HTA in Iran, and it should be a part of the government's agenda to manage its limited resources effectively and provide a more appropriate response.

## Data Availability

Not applicable.

## Abbreviations

HTA: Health technology assessment
UHC: Universal health coverage
MoHME: Ministry of Health and Medical Education
HTP: Health transformation plan
